# An Evaluation of a Parenteral Antibiotic Treatment of Cattle with Non-Healing Claw Horn Lesions

**DOI:** 10.3390/ani14101396

**Published:** 2024-05-07

**Authors:** Menno Holzhauer, Siert-Jan Boersma, Dorien Boon, Han de Leeuw

**Affiliations:** 1 Ruminant Health Department, Royal GD-Animal Health, P.O. Box 9, 7400 AA Deventer, The Netherlands; 2 Veterinary Practice De Rijp, Schoener 2, 1483 TP De Rijp, The Netherlands; siert-jan.boersema@evidensia.nl (S.-J.B.); dorien.boon@evidensia.nl (D.B.); 3 Private Claw Trimmer, Krommeniedijk, 134 1562 GP Krommenie, The Netherlands; j.leeuw986@upcmail.nl

**Keywords:** lameness, toe necrosis, non-healing white line disorder, welfare, durability

## Abstract

**Simple Summary:**

Lameness in dairy cattle is mainly caused by affection of the under feet. This affection can be caused by an infection of the skin (e.g., digital dermatitis or interdigital phlegmon) or by non-infectious claw horn lesions (e.g., sole ulcers, white line lesions, or toe necrosis). The latter of these are sometimes infected secondarily by environmental bacteria like *Spirochetes,* and those lesions were for many years considered to be non-healing and responsible for the premature culling of dairy cows. In a clinical pilot study with a one-group post-test-only design, dairy cows with such infected claw horn lesions were claw trimmed, topically treated, and additionally injected with tilmycosin parenterally. A follow-up after 3 months showed not only a better cure, but also an almost normal locomotion and better production, even in comparison with non-affected herd mates.

**Abstract:**

Non-healing claw horn disorders are a serious problem in dairy herds because of the long duration of the disorder and the chronic pain derived from it, seriously affecting animal welfare and causing decreased production and premature culling from the herd. In a clinical trial, 40 cows in 13 herds (12 dairy herds and 1 herd with cow–calf operations) with toe necrosis (TN, 27x) or a non-healing white line disorder (NHWLD, 13x) were treated topically with an unguent-containing zinc sulphate and acetyl acid in combination with a parenteral injection of tilmycosin. An evaluation was conducted 3 months after treatment using locomotion scoring (LS), a clinical observation of the lesion, and the lactation value (the lactation value is the Net Profit of the individual animal divided by the average Net Profit of the entire herd. The mean is 100, so >100 is related to better production (combination of kg milk, %fat, and protein)) (LV) before and after treatment. The mean LS improved significantly from 4.0 (SD: 0.2) before treatment to 1.2 (SD: 0.4) 3 months after treatment (*p* < 0.001). The clinical presentation showed that all of the cows were cured from horn shoe infection (both TN and NHWLD). The LVs of the treated cows increased significantly from 111.2 (SD: 12.2) to 116.8 (SD: 15.1; *p* = 0.003).

## 1. Introduction

Lameness in dairy cattle is, alongside mastitis and decreased fertility, a serious herd-related problem, responsible for decreased animal welfare due to pain and the prolonged existence of the disorder, as well as economic losses due to, among others, reduced production, premature culling, and a loss of body weight at slaughter [1,2,3]. The latter is mainly a consequence of decreased milk production, extra labor, and premature culling from the herd [4,5,6,7]. A Danish investigation concluded that locomotor disorders were the reason for approximately 40% of all cows being euthanized, making them the most frequent reason for on-farm euthanasia of Danish dairy cows [8]. Recently, a benchmarking of claw health was introduced for the comparison of one’s own farm’s claw health with a large number of other dairy farms with similar performance levels. This benchmarking may further support the analysis of the improvement potential of one’s own farm, may encourage farmers and veterinarians to improve animal welfare, and may be helpful for minimizing economic losses due to lame cows [9].

The cause of lameness has its origin mainly in the hoof and can be both of an infectious and non-infectious origin. All infectious claw disorders are related to each other and are normally related to an affection of the skin around the hoof and in the interdigital space. Non-infectious disorders are normally a consequence of an affection of the horn shoe itself [10]. The most frequently noticed infectious lesions are digital dermatitis (DD), with a herd prevalence of about >90% and a cow prevalence of 20–25% in The Netherlands [11], and interdigital phlegmon (IP), with a low herd prevalence and an average cow incidence of 10–12%. Digital dermatitis is associated with bacterial infections like *Treponema spp.,* and IP is especially associated with *Fusobacterium necrophorum*. The most frequently noticed non-infectious claw disorders or claw horn disruption lesions (CHDLs) are white line disorders and sole ulcers, with prevalences in The Netherlands of, respectively, 18% and 9% [12]. Comparable data were estimated in other Western European countries like the UK and Switzerland [13,14].

In all these countries, but also in other European countries and, e.g., North America also, some CHDLs, including toe necrosis (TN), are considered non-healing, and cows may have these lesions for several months, and sometimes for more than a year [15,16]. This means that despite intensive standard treatment (claw trimming and topical application of any spray or unguent), a cure cannot be realized [17,18,19]. Toe necrosis (TN) is related to an osteitis, and a non-healing white line (NHWLD) is related to infection of the pododerma in the absence of osteitis [20]. In recent years, the proportion of NHWLDs has increased from 1–2% to 10% in some herds (according to the personal experience of the authors). Due to the non-healing aspect, most patients experience great discomfort (chronic lameness and pain) and are frequently involuntarily culled [21]. The problems are considered to be caused by secondary bacterial infections like *Treponema* spp. and *Porphyromonas endodontalis* [22]. Due to this involvement, these lesions are currently classified as a DD-associated white line abscess (DD-WLA) and DD-associated toe necrosis (DD-TN) [23].

In recent years, the only treatments that were applied were surgical intervention of the affected toe tip [24] and the removal of deviated horn under local anesthesia with the help of a grinder [25]. The surgical intervention of the toe tip is still used but is not suitable for DD-WLA because of a difference in pathogenesis, e.g., affection of the pododerma, including the pedal bone (DD-TN) and affection of the pododerma (DD-WLA). In the UK, a 5-day treatment with antibiotics like ceftiofur hydrochloride was identified as successful [15], but this antibiotic is no longer registered for dairy cattle in the Netherlands. Ceftiofur belongs to the group of third-generation cephalosporines and has bactericide characteristics. The involvement of a bacterial infection in this type of disorder was clear, and the researchers participating in this project experienced a remarkably successful treatment of these disorders with an additional subcutaneous injection with tilmycosin. The objective of this study was to evaluate the effect of protocollary claw trimming by a certified claw trimmer, combined with a single parenteral injection with tilmycosin. The evaluation was based on locomotion, a clinical evaluation of the claw horn capsule, and the presence or absence of a typical smell and a derivative production parameter.

## 2. Materials and Methods

This study was designed as a one-group post-test-only design, whereby the effect of the intervention was evaluated by clinical observation and progress on locomotion on the one hand and improvement in a derivative production parameter in comparison with non-affected herd mates within the same herd on the other hand. All procedures were approved by the Royal GD Animal Health ethics committee.

**Figure 1:**
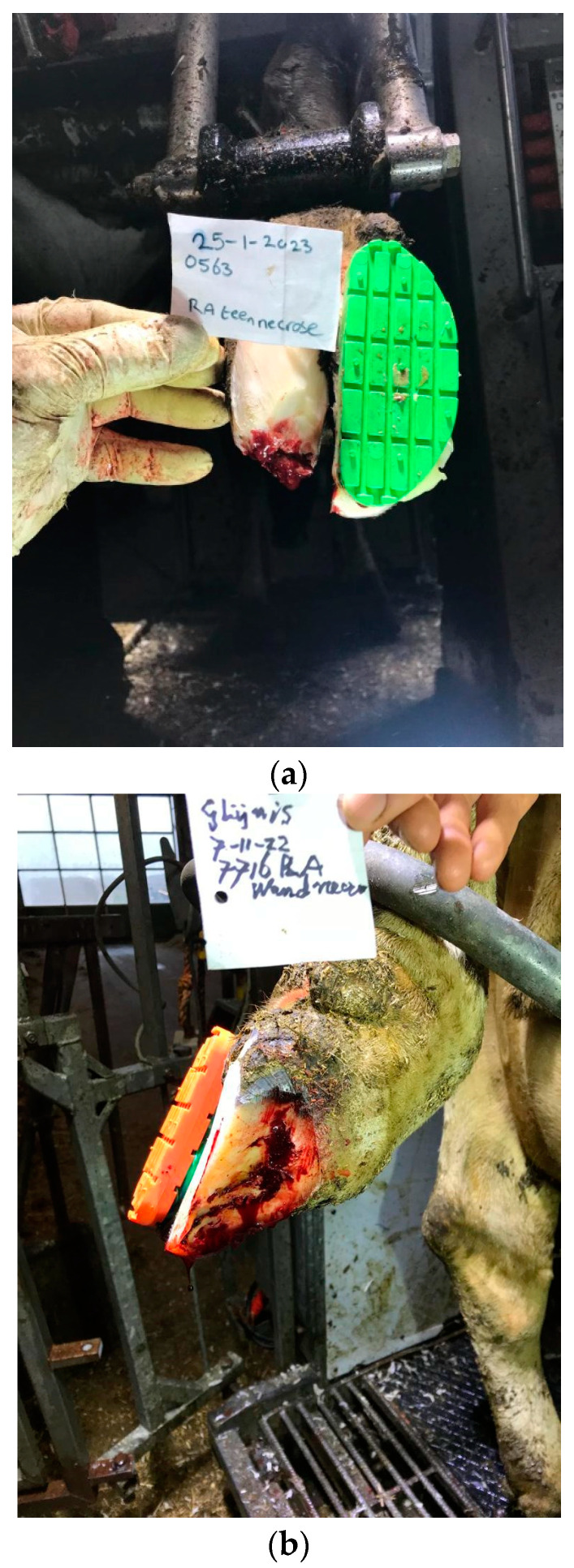
(**a**) DD-infected toe necrosis before topical treatment with an unguent and parenteral tilmycosin injection. (**b**) DD-infected white line disorder before topical treatment with an unguent and parenteral tilmycosin injection.

The cows in this study belonged to the customer base of the Veterinary Practice De Rijp and were visited by Sir Han de Leeuw (HdL) for preventive and curative claw trimming. All farms included in the study except one were dairy farms. The cows included in the project were suffering from DD-TN or DD-WLA (see Figure 1), and the lesions were in line with the definitions in the ICAR Claw Health Atlas—Appendix A Digital Dermatitis-Associated Claw Horn Lesions [23].

All cows were trimmed and lesions treated at least twice according to the standard Dutch 5-step method [26].The Dutch 5-step method included estimating the length of the inner claw (ideal: 7.5 cm) and, when necessary, shortening the horn shoe and leveling the sole surface (1); ensuring that the outer claw was of the same length and as high as the inner claw, if possible (2); modeling by slight removal of the axial wall horn, until 2.5 cm from the tip of the claw (3); lowering of the sole of the affected claw (4); treatment of present lesions (5); and checking of the coronary band and interdigital space for lesions and treating any lesions present [26]. In addition, the cows with DD-WLA and DD-TN were treated at least twice according to the same standard treatment of removal of all deviated horn, topical application of an unguent, a block on the other claw of the same leg, and a parenteral NSAID injection before the study. The applied standard treatment for the cows in the study included standard claw trimming, removal of all of the loose horn, a hard plastic block under the contralateral claw of the same leg, topical treatment with an unguent which consisted of zinc sulphate, salicylic acid, and some trace elements dissolved in tar, under a bandage, and a parenteral treatment with an NSAID and a single tilmycosin injection subcutaneously. In this pilot study, we chose a plastic block because these blocks hardly ever wear out on a concrete floor, unlike wooden blocks that often wear unevenly, which can disrupt an animal’s gait or weight distribution, resulting in persistent lameness. Typically, rubber blocks are more durable than wooden blocks, and on average, rubber blocks stay adhered on claws for more than six weeks after application. However, rubber blocks usually require a hoof care professional to remove them once an injury heals [27]. The bandage was always removed on day 5 by the farmers. Conditions for inclusion were that the last topical treatment had been administered more than 1 month before the day of the intervention with tilmycosin, and that milk production data were available as one of the objective parameters. Other parameters were a visual evaluation of the healing of the lesion and a locomotion score. Based on the sizes of the lesions (1–1.5 cm) and a normal horn growth of 5–6 mm/month [10], the lesions were evaluated before and 3 months after treatment. All cows were locomotion-scored in the building for at least 4–6 steps according to Sprecher et al. [28] (see Table 1) before the start of the trimming on the same floor and before applying a block and at 3 months after removal of the block. The lesions were evaluated based on the presence of penetration of the horn capsule and a very typical necrotic smell, and they were photographed before application of the unguent by the claw trimmer and also at the moment of evaluation of the lesion (HdL).

### Statistical Evaluation

The locomotion score and the milk production in comparison to herd mates (lactation value, which is the Net Profit of the individual animal divided by the average Net Profit of the entire herd) at the time of intervention and at the time of evaluation were recorded. For data analysis, a sign test was used in the statistical program IBM Stata v.15.1 (StataCorp, College Station, TX, USA, 2017).

## 3. Results

**Figure 2:**
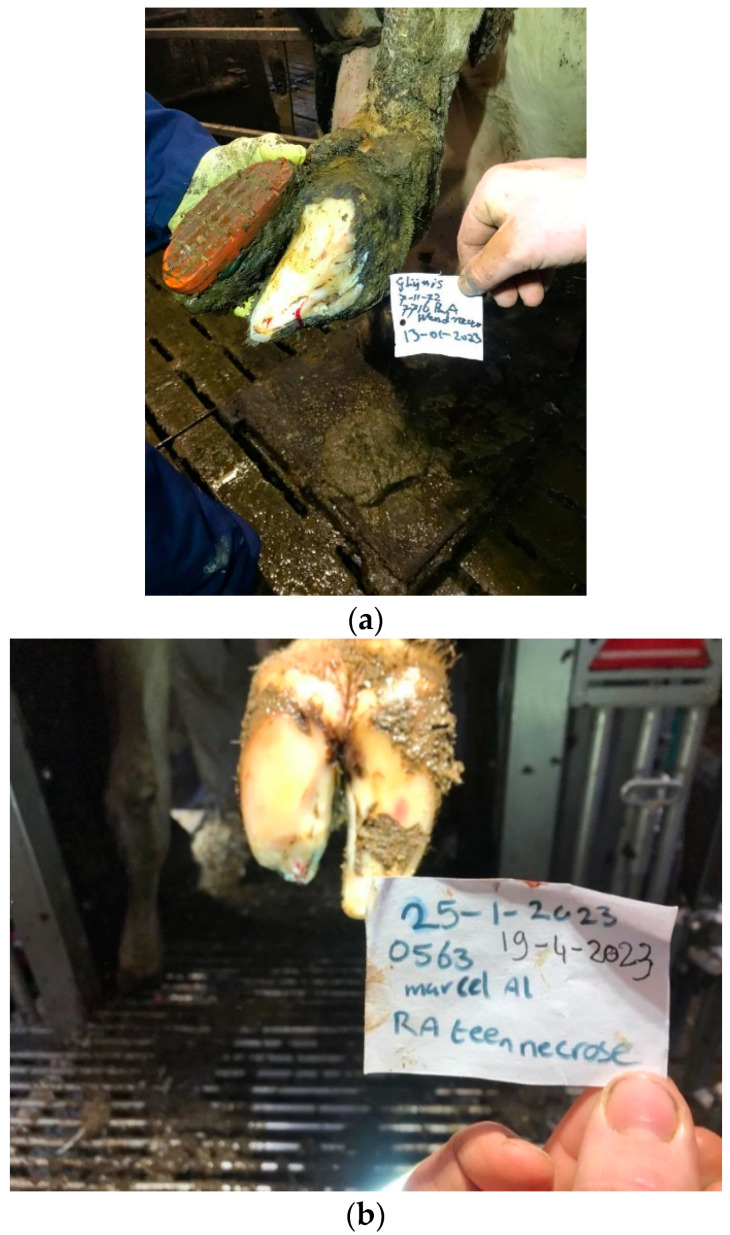
(**a**) DD-infected toe necrosis and 3 months after topical treatment with an unguent and parenteral tilmycosin injection. (**b**) DD-infected white line disorder 3 months after topical treatment with an unguent and parenteral tilmycosin injection.

The intervention was performed on 40 cows in 13 herds (12 dairy and 1 with cow–calf operations). The mean herd size was 179.5 cows (SD = 108.6), and the mean milk production of cows in the dairy herds was 9367 kg/305 days (SD: 729.7). The mean age of the cows in the study was 4.9 years (SD: 1.8), and 32 were Holstein Friesian, 4 Montebeliard, and 4 beef cattle (mixed-breed). Twenty-seven cows (eight inner claws) were treated for DD-TN, and thirteen (all outer claws) were treated for DD-WLA. In total, 37 cows were evaluated 3 months after treatment, 1 cow was culled for clinical mastitis, and 2 cows had missing data from the milk-recording list. Two youngstock with affected dewclaws received the standard treatment but were excluded from the evaluation. The clinical presentation at 3 months showed that all the cows were clinically cured of the infestation of the horn shoe. This meant that there was at the moment of evaluation no indication of penetration of the horn capsule (both DD-TN and DD-WLA, see Figure 2), no pain at palpation of the healing lesion, and an absence of the typical necrotic smell. The mean LS of the cows included was 4.0 (SD: 0.2) before treatment and 1.2 (SD:0.4) at 3 months after treatment and before application and after removal of the hard plastic block, respectively. The lactation value of the treated cows improved in the 3-month period from 101.3 (SD: 14.6) to 108.7 (SD: 15.2). Both improvements were statistically significant (*p* < 0.001 and 0.003, respectively).

## 4. Discussion

As far as we know, in this project, we have shown for the first time that a topical treatment with an unguent based on salicylic acid, zinc sulphate, and some trace elements dissolved in tar combined with a single parenteral injection with tilmycosin resulted in a complete cure in >95% of the cases. Thus, it seems to be a good alternative for other types of interventions like partial claw amputation under local anesthesia in case of DD-TN or removal under local anesthesia with a grinder or surgical removal of the tip of the claw [24,25]. The cure can theoretically also be the consequence of repeated topical treatment, but it is the investigators’ experience that a cure based on intensive topical claw trimming treatment is exceptional (<1–2%). Practitioners do not execute claw trimming in our country on a regular basis. Most of them simply do not have enough experience. Besides that, there is enough other work for them to do before the end of the day. That was already the experience of the farmers before the start of this pilot study, and therefore, they were not prepared to participate in a case–control study and to have their cows with chronic DD-TN and DD-WLA lesions treated topically only with a comparison with a parenteral treatment with tilmycosin, as planned in the original design of the project. Consequently, there was no control group with a hard plastic block under the contra-lateral claw of the same leg and parenteral NSAID, since we expected that this would not result in the desired cure. The limitation of this study was the absence of a real control group, because the farmers were not motivated (1) to participate in this study design. One could also say that the cows were actually their own control group; housing in the same environment, feeding the same ration, and just topical treatment did not result in any cure of one of the types of lesions investigated (2). Of course, the potential weaknesses of the research are that the patients may have received special attention and/or were unintentionally treated differently. It is advisable to repeat the study with control animals and blindness of the treatment for the farmers.

In this study, all cattle were checked for the presence or absence of penetration of the horn capsule, presence of pain upon touch, presence of the typical smell of necrotic tissue, and both locomotion scores [28] and lactation values at 3 months, which is not a very long period, but in line with what could be expected based on the size of the lesion and normal growth of claw horn [10]. From previous experience, we know that recurrence of these lesions is highly exceptional. Both the locomotion and the lactation values were evaluated as objective parameters by considering improvement, and these were significant. For the farmers, it was most important that the cows could take better care of themselves in the herd and that they no longer behaved differently due to the lameness being solved (better welfare). Normal behavior with regular feed intake also improved milk production significantly in relation to the herd mates, who received the same ration, and this was expressed in the lactation value. The most important improvement in the farmers’ experience was better animal welfare and that irritation and time spent on extra animal care disappeared.

DD-TN and DD-WLA are both claw disorders that frequently result in premature culling from the herd despite intensive (monthly) claw trimming and topical treatment. The prevalence of both disorders in the Netherlands is based on Digiklauw, a Dutch data recording system for claw disorders and is currently 1–2%, with outliers of up to 10% [12,29]. The average size of the herds in this study was 179.5 (SD = 108.1), which was larger than the average Dutch dairy farm (*χ* = 104.8; Dutch Central Office for Statistics, 2023). Ninety-two percent of the cows in this study were housed in free stalls on slatted floors, and the remaining cows were housed on partly solid concrete floors and other housing systems such as straw yards, which is comparable with the Dutch population of dairy herds [30]. A study in the UK on risk factors for increased rates of sole ulcers, white line disease, and digital dermatitis in dairy cattle showed that the risks for white line disease increased with an increasing parity and herd size, cows being on pasture by day and housed at night, and solid grooved concrete floors in yards or alleys [13]. The larger herds in this study may be an explanation for the higher risk of white line lesions, and TN frequently starts as an axial white line lesion [18]. Exact information about pasturing and whether floors were grooved or not was not available. Additionally, if so, that does not explain the presence of DD-TN and DD-WLD. The risk factors for the occurrence of that presumed secondary infection are unknown, while DD is found in >90% of the herds in our country, and the presence of M2 lesions is not higher than it used to be (based on personal observations).

Infection of the pedal bone in horses (pedal osteitis, PO) is a common complication of external trauma or laminitis, and most of the time, it is seen in the forelimbs. Based on radiographic investigation, the signs are related to the demineralization, widening of the nutrient foramina at the solar margin, and irregular bone formation along the solar margins of the distal phalanx. Treatment aims to reduce the inflammation within the distal phalanx, minimize damage to the foot, and eliminate the inciting cause. Treatments are mainly corrective shoeing, oral NSAIDs, and rest. The treatment of septic PO usually involves systemic and local antimicrobials (regional limb perfusion) and surgical debridement of the infected bone. Progress is mainly based on radiographic investigation [31]. In other species, osteitis is to a larger extent a part of an osteomyelitis problem, with *Staphylococcus aureus* being the most prevalent causal organism, and researchers are looking for topical application of antibiotics in particular [32]. In human medicine, bone and joint infections are experienced as painful for patients and frustrating for both those who suffer from it, as well as their doctors. The high success rates of antimicrobial therapy in most infectious diseases have not yet been achieved in bone and joint infections due to the physiological and anatomical characteristics of bone. The key to successful management is early diagnosis, including bone sampling for microbiological and pathological examination, to allow for targeted and long-lasting antimicrobial therapy [33]. 

Indications for the use of antibiotics in the case of claw horn disorders are very limited. The only well-known indication for the systematic use of antibiotics is acute lameness causing interdigital phlegmon [34,35]. For digital dermatitis, different antibiotic-based products are licensed to be used for topical treatment [36], although today, different non-antibiotic products have been proven and are also licensed [37,38,39]. Thus, in the authors’ experience, both of the investigated claw disorders, DD-TN and DD-WLA, are, besides interdigital phlegmon, the only lesions that justify the parenteral use of antibiotics.

The research team is well aware that antibiotics should be used restrictively [40], but the results presented here are good enough that the correct use in comparable cases is justified, since no other treatments with comparable results are known in bovine medicine. DD-affected claw horn lesions are very painful, and healing is important for better durability, welfare, and economic results in the dairy industry.

## 5. Conclusions

In a pilot study, 27 cows with DD-TN and 13 cows with DD-WLD were claw trimmed, topically treated with an unguent, and an parenteral injection of an NSAID and tilmycosin. At an evaluation at 3 months, the clinical healing, the locomotion, and the lactation value (an objective production parameter for cows within the same herd) showed a clinical cure in >90% of cows and significant improvements in the locomotion and the milk production. Consequently, a single additional injection with tilmycosin seems to be an alternative therapy in these cases.

## Figures and Tables

**Table 1:** Lameness scoring according Sprecher et al. (1997).

Lameness Score	Clinical Description	Assessment
1	Normal	The cow stands and walks with a level-back posture. Her gait is normal.
2	Mildly lame	The cow stands with a level-back posture but develops an arched-back posture when walking. Her gait remains normal.
3	Moderately lame	An arched-back posture is evident both while standing and walking. Her gait is affected and is best described as short-striding with one or more limbs.
4	Lame	An arched-back posture is always evident, and the gait is best described as one deliberate step at a time. The cow favors one or more limbs/feet.
5	Severely lame	The cow additionally demonstrates an inability or extreme reluctance to bear weight on one or more of her limbs/feet.

**Figure A1:**
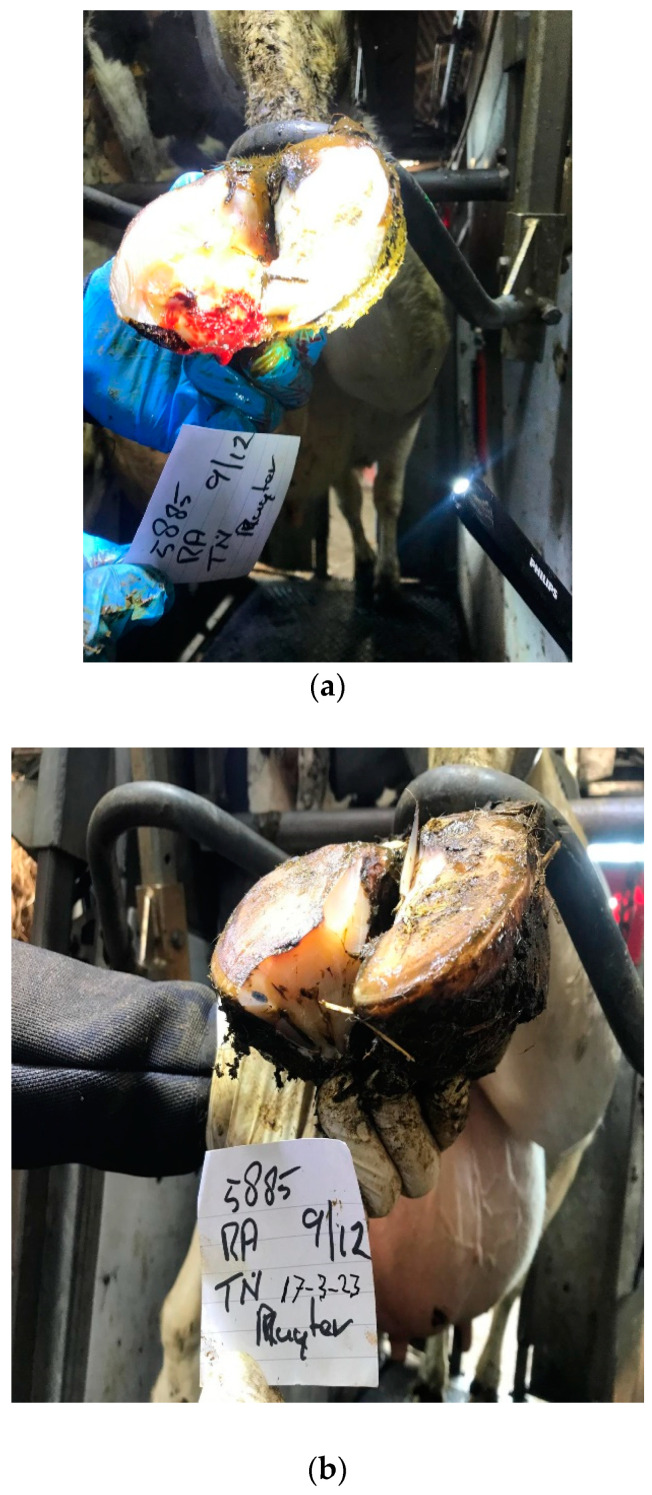
(**a**) DD-infected toe necrosis before topical treatment with an unguent and parenteral tilmycosin injection. (**b**) DD-infected toe necrosis and 3 months after topical treatment with an unguent and parenteral tilmycosin injection.

**Figure A2:**
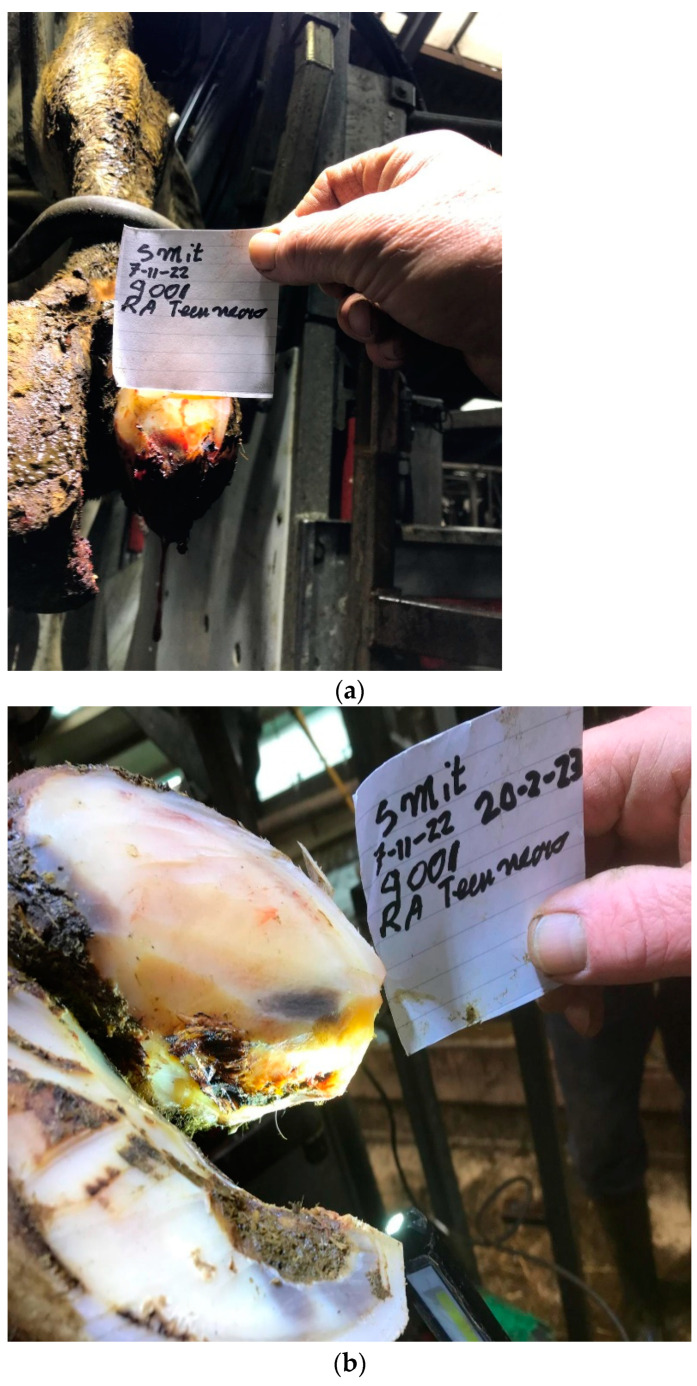
(**a**) DD-infected toe necrosis before topical treatment with an unguent and parenteral tilmycosin injection. (**b**) DD-infected toe necrosis and 3 months after topical treatment with an unguent and parenteral tilmycosin injection.

**Figure A3:**
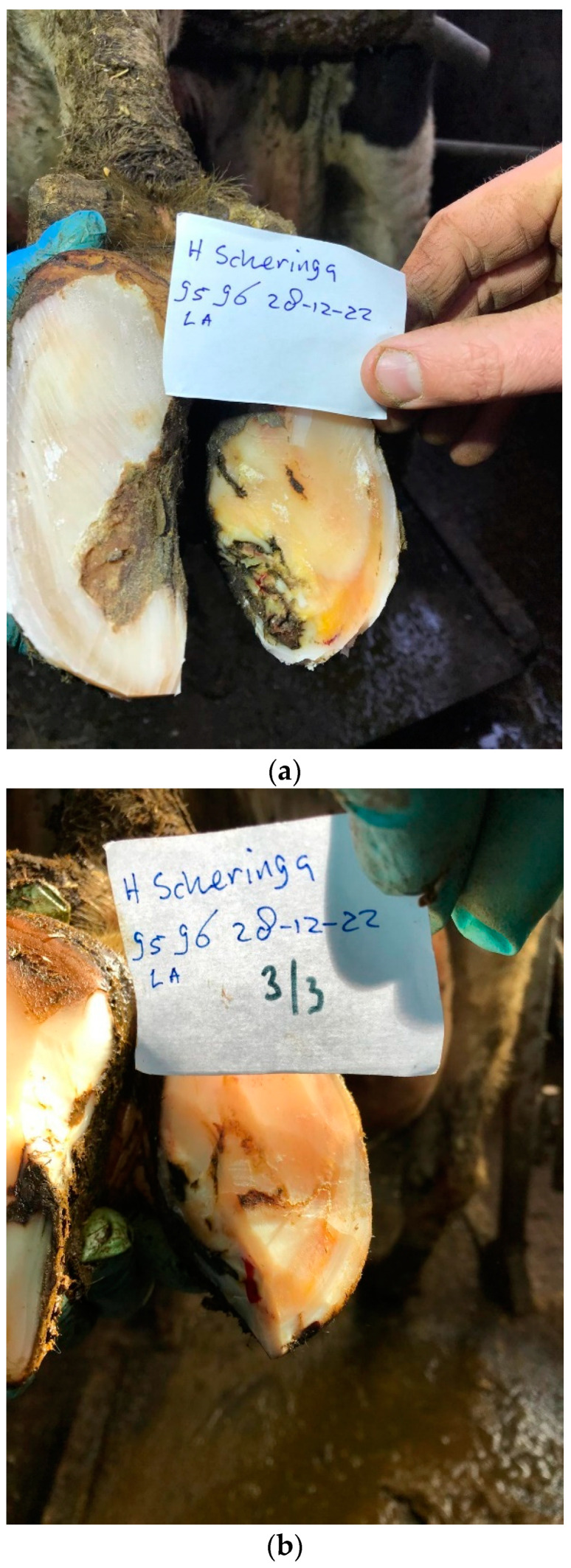
(**a**) DD-infected toe necrosis before topical treatment with an unguent and parenteral tilmycosin injection. (**b**) DD-infected toe necrosis and 3 months after topical treatment with an unguent and parenteral tilmycosin injection.

**Figure A4:**
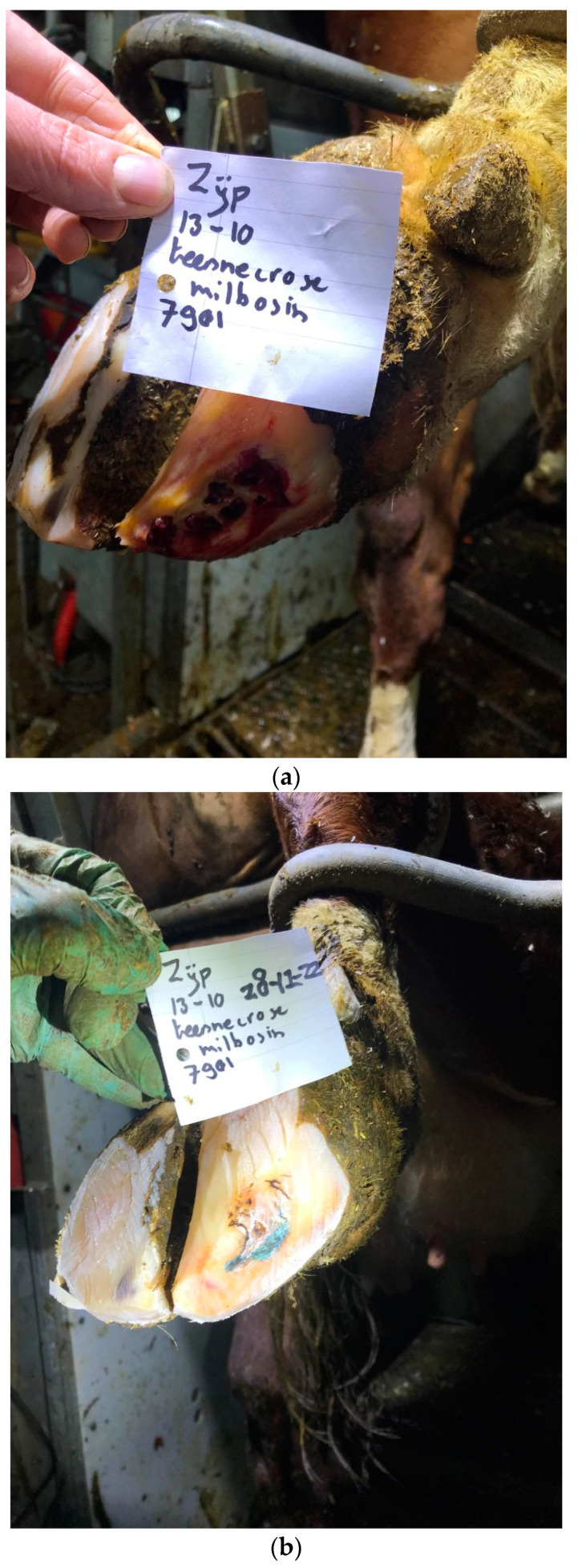
(**a**) DD-infected toe necrosis before topical treatment with an unguent and parenteral tilmycosin injection. (**b**) DD-infected toe necrosis and 3 months after topical treatment with an unguent and parenteral tilmycosin injection.

**Figure A5:**
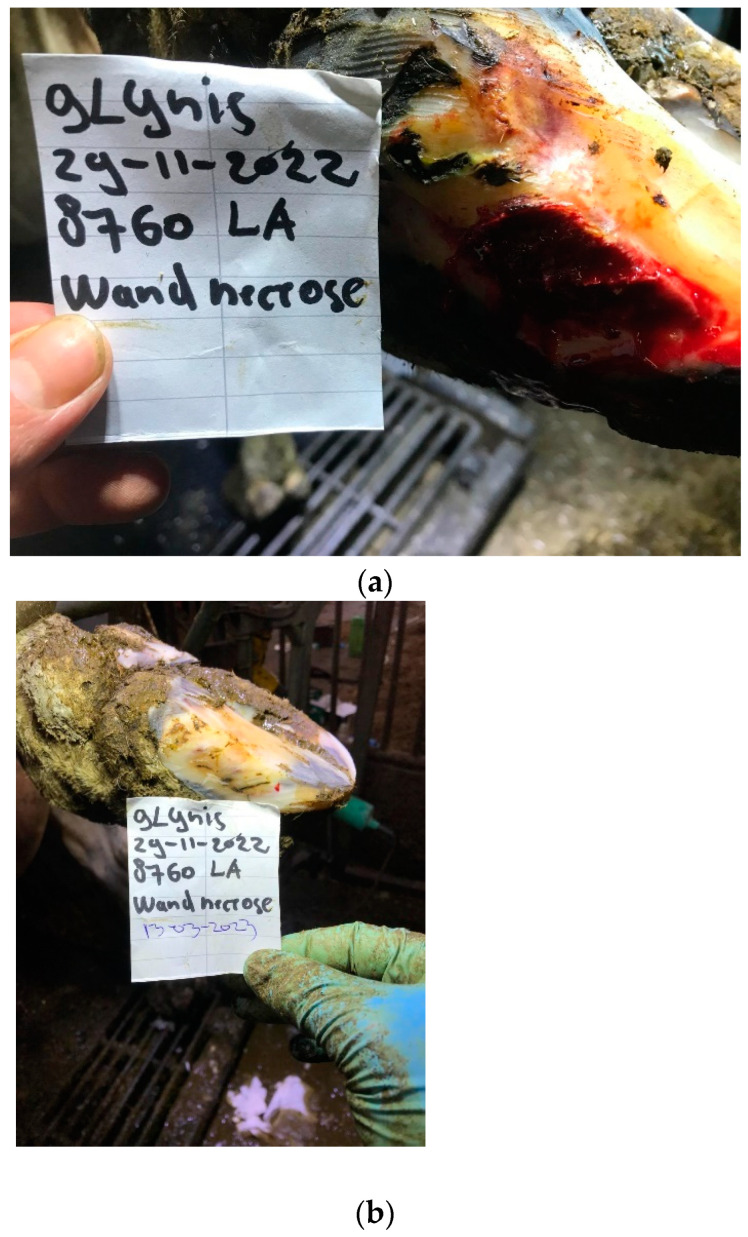
(**a**) DD-infected white line disorder before topical treatment with an unguent and parenteral tilmycosin injection. (**b**) DD-infected white line disorder and 3 months after topical treatment with an unguent and parenteral tilmycosin injection.

**Figure A6:**
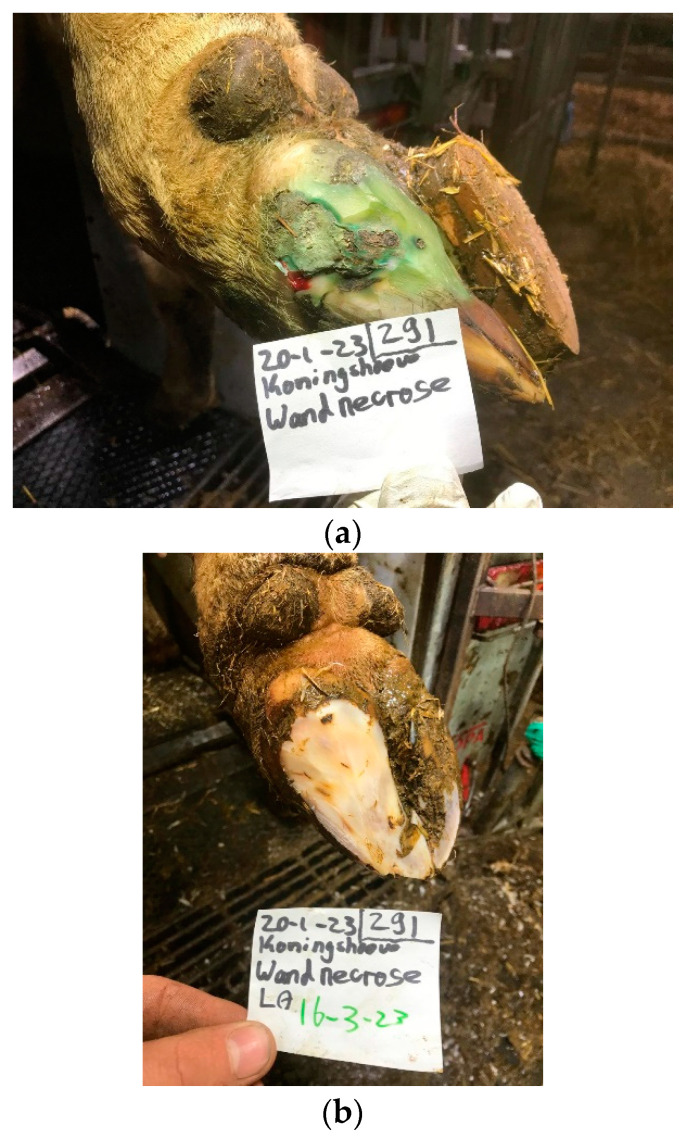
(**a**) DD-infected white line disorder before topical treatment with an unguent and parenteral tilmycosin injection. (**b**) DD-infected white line disorder and 3 months after topical treatment with an unguent and parenteral tilmycosin injection.

**Figure A7:**
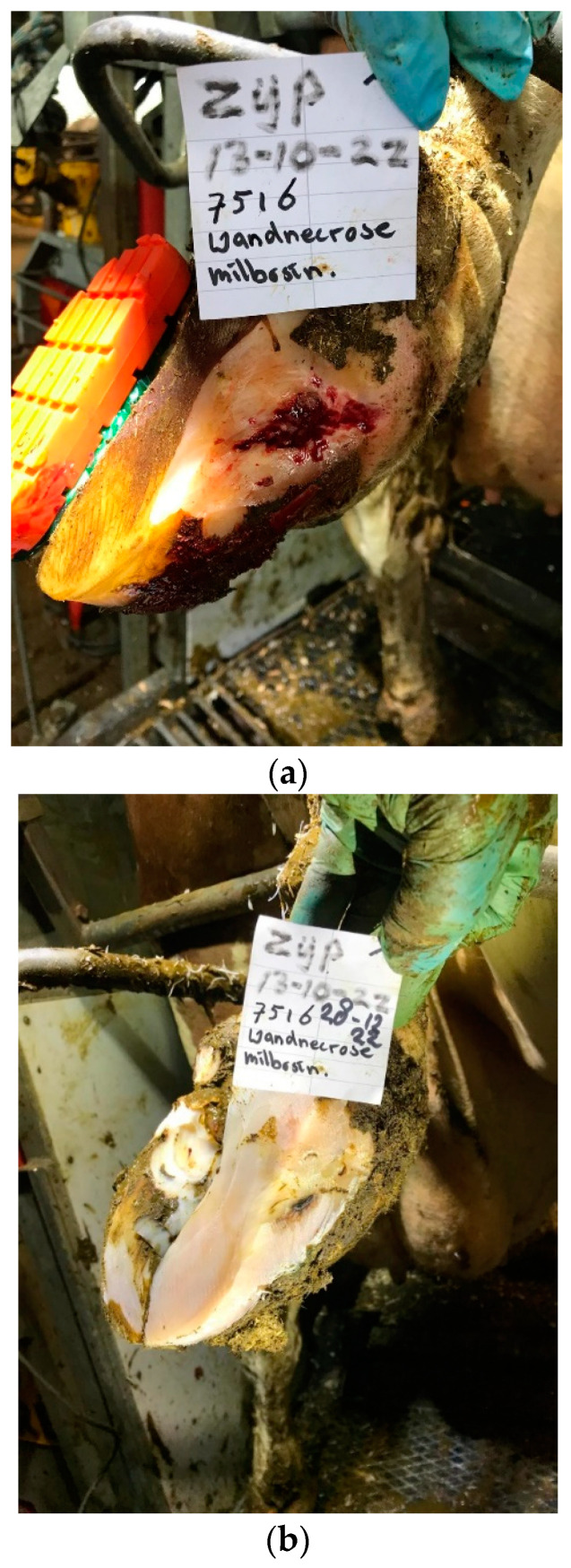
(**a**) DD-infected white line disorder before topical treatment with an unguent and parenteral tilmycosin injection. (**b**) DD-infected white line disorder and 3 months after topical treatment with an unguent and parenteral tilmycosin injection.

## Data Availability

All data are available from the first author on request.
